# Adequate Food and Nutrition in School: Acceptability and Consumption by Students of a Brazilian Municipality

**DOI:** 10.3390/nu17030528

**Published:** 2025-01-31

**Authors:** Patricia Henriques, Camile R. T. de Alvarenga, Marina M. S. Menezes, Daniele M. Ferreira, Luciene Burlandy, Beatriz D. Soares

**Affiliations:** Programa de Pós-Graduação em Ciências da Nutrição, Universidade Federal Fluminense, Niterói 24020-140, Brazil; camiletorres@id.uff.br (C.R.T.d.A.); marinamessas@id.uff.br (M.M.S.M.); daniele_ferreira@id.uff.br (D.M.F.); lucieneburlandy@id.uff.br (L.B.); beatrizds@id.uff.br (B.D.S.)

**Keywords:** childhood obesity, public health, school-based interventions

## Abstract

Objective: This study aimed to evaluate the acceptability of a specific menu preparation based on minimally processed foods and the factors associated with the consumption of school meals by students in Brazilian public schools. Methods: A hedonic scale was employed to assess the acceptability of a milk preparation with 100% cocoa and sugar. A structured questionnaire was used to evaluate school food consumption, addressing consumption frequency, dietary habits, environmental conditions, and consumption of competing foods. A binomial logistic regression model was conducted to verify the association between individual variables and conditions of meal supply and consumption. Data analyses were performed using the Jamovi 2022 software. Results: A total of 1080 students participated, with 630 taking part in the acceptability test, and 450 completing the consumption questionnaire. The study highlighted acceptance below the minimum PNAE standards (79.68%) for the tested preparation, indicating a need for further investigation into student preferences. Pleasant taste was the most frequently cited reason (53.2%) for consumption. Disliking the served lunch increased the likelihood of not consuming school meals by 2.73% (*p* = 0.03). Bringing snacks from home showed no significant impact on consumption (*p* = 0.677). Using inadequate cutlery increased the likelihood of not consuming school meals by 6.44% (*p* = 0.009). Conclusions: The study underscored the low acceptance of milk prepared with 100% cocoa and sugar, along with irregular school meal consumption, emphasizing the need for strategies to align students’ taste preferences with healthier menu options. While PNAE ensures nutrient-rich meals, improving flavor, variety, and dining conditions, alongside permanent food and nutrition education, is essential to enhance adherence and support students’ health. Although snacks do not affect the consumption of school meals, controlling snack intake is important from a health perspective.

## 1. Introduction

Adequate nutrition during school-age years is fundamental for maintaining proper growth and development, addressing deficits of undernutrition, correcting nutritional inadequacies, and mitigating the longer-term consequences of obesity [1]. During this period, the food provision supplies essential nutrients and serves as a strategy to promote healthy eating habits [2], which makes this period highly relevant, considering eating preferences and behaviors [3].

In this sense, school age is the most significant nutritional demand stage and is related to energy repletion with energy storage for the period that includes the puberty spurt [4]. This is when appetite increases, there is better acceptance of food, greater food choice autonomy, and increased influence of the social group on food choice. At this stage, along with the family, schools play a crucial role in promoting children’s health [5].

In Brazil, the National School Meal Program (PNAE) is an essential policy in guaranteeing food and nutritional security for students in the public education network, considering its universal, equitable, participatory, integrative, sustainable, and healthy nature [6]. The PNAE aims to provide balanced and quality meals to primary education students, complementing their nutritional needs during the school period. Therefore, it contributes to growth, development, learning and school performance, and the establishment of healthy eating habits [7].

Recognizing the school environment as a strategic space for promoting healthy eating, the PNAE aligned its 2020 guidelines with national dietary guides (for the Brazilian Population and Children Under Two Years of Age) [8,9], resulting in new nutritional recommendations for students, especially the obligation that 75% of resources must be allocated to the acquisition of fresh or minimally processed foods, a maximum of 20% to the acquisition of processed and ultra-processed foods (UPF), and a maximum of 5% for the acquisition of processed culinary ingredients [6].

Minimally processed foods are natural foods that have undergone small changes for preservation or preparation, such as washing, peeling, freezing, or pasteurization, without adding substances that significantly alter their nutritional profile. Examples include fresh fruits, vegetables, and frozen meats. Processed foods are those modified by the addition of salt, sugar, or other culinary ingredients to enhance flavor or shelf life, such as canned vegetables or cheeses. UPF, on the other hand, are industrial formulations made mostly from substances extracted from foods (e.g., oils, sugars) and additives like flavorings, colorants, and emulsifiers, which are designed to be convenient and appealing but often lack nutritional balance. Examples include soft drinks, packaged snacks, and instant noodles [8]. Replacing less healthy foods with more nutritious options, such as fruits and vegetables, is an important strategy to ensure adequate nutrition for students. This approach supports both physical and cognitive development, as evidence suggests that early exposure to UPF may have negative effects on children’s nutritional status [10].

A scoping review highlighted the consistent association between UPF consumption and obesity, or indicators related to it, in adults, with studies showing a dose–response relationship in cross-sectional research from five countries [11]. Similarly, a meta-analysis identified that UPF consumption is associated with an increased risk of developing overweight, body and abdominal obesity, and more specifically with dyslipidemia in children and metabolic syndrome in adolescents [12].

In this context, a systematic review that analyzed the relationship between school food environments, individual food consumption patterns, and excess weight in South American schoolchildren identified school environment factors from the policy and physical domains, such as unsatisfactory food and nutrition education and unavailability of school-prepared meals, were associated with increased prevalence of excess weight. Individual factors related to adherence to the school meal program, such as the consumption of meals offered by the school instead of bringing a snack from home, were associated with a lower prevalence of excess weight [13].

From this perspective, promoting adequate and healthy eating in the school environment is an important strategy for preventing and reversing childhood obesity, and is widely recommended by international organizations [14,15,16]. However, although the PNAE offers an adequate and healthy diet with health-promoting attributes, the low frequency of consumption of school meals has been evidenced in the literature [17,18,19,20,21,22]. This issue is attributed to several factors, especially the consumption of competitive foods—UPF and other foods sold inside and around public schools—that can affect students’ food choices [23,24,25].

The preference for foods with low nutritional value at the expense of school meals can affect the health of children and adolescents, such as developing nutritional deficiencies, obesity, and greater susceptibility to chronic non-communicable diseases [26,27,28]. In this sense, the school context effect has been identified as a determinant of school food consumption, especially when considering factors such as taste, environment, and how meals are served, which can directly influence the acceptability, frequency, and consumption pattern of school meals [29,30].

The offering of preparations based on minimally processed foods, such as milk with 100% cocoa and sugar, as evaluated in this study, aligns with the principles of promoting healthy eating in the PNAE and is an important strategy for reducing the consumption of UPF. Therefore, our hypothesis is that the acceptability of this specific menu preparation is inadequate and that the food supply conditions at school may influence its consumption. Thus, this study aimed to evaluate the acceptability of a specific menu preparation based on minimally processed foods and the factors associated with the consumption of school meals by students in Brazilian public schools after the publication of legislation that restricts the supply of unhealthy foods in school meals [6].

In the present study, acceptability was defined as the acceptance index of the foods offered to schoolchildren, considering their sensory perception of characteristics such as flavor, texture, and appearance. Acceptability is a crucial factor in determining whether school meals are consumed and appreciated, directly influencing the success of initiatives like the PNAE in promoting healthy eating habits and minimizing food waste [31].

Despite the well-documented benefits of school meal programs like the PNAE in promoting healthy eating habits and addressing childhood obesity, there is limited research exploring the multifaceted factors influencing the acceptability and consumption of school meals, especially in the context of recent legislative changes aimed at reducing ultra-processed food consumption. The existing literature insufficiently addresses how individual preferences, school food supply characteristics, and the broader school environment interact to shape food choices within this setting. Furthermore, local studies investigating adherence to PNAE guidelines and the impact of competitive foods on meal consumption are scarce, leaving critical gaps in understanding region-specific challenges and opportunities.

This study addresses these gaps by analyzing several aspects related to the consumption of school meals in public schools, considering individual issues, characteristics of the food supply, conditions of the school environment, and the presence of competitive foods. The results can help to understand the factors that influence the consumption of school meals that are poorly explored in the scientific literature.

In addition, local research on adherence to the PNAE is crucial for identifying specific challenges [32], tailoring interventions, engaging the community [33], and improving monitoring and evaluation processes [34]. Such localized insights can improve the effectiveness and sustainability of national programs. This, in turn, contributes to improved educational and health outcomes among children [33], particularly through implementing strategies to prevent and manage childhood obesity.

## 2. Materials and Methods

### 2.1. Study Design and Participants

This cross-sectional, quantitative study is nested in the “Study of the Food Environment, the Acceptability of Menu Preparations, and the Factors Associated with Adherence to School Meals in Public Schools in the State of Rio de Janeiro”.

The study population included first-to-fifth graders (6–10 years old) of elementary schools in the municipal education network of a city in the State of Rio de Janeiro (Brazil), enrolled in seven schools selected to represent the municipal education centers. Simple random sampling was employed with a 5% margin of error and a 95% confidence level. The sample calculation was performed using the equation: *n* = [N.Z2.p(1 − p)]/[((N − 1.e2) + (Z2.p.(1 − p))], where *n* = sample size; N = total first-to-fifth graders enrolled in the municipal elementary school in 2021 (5078); Z = z-score (1.96) for the 95% confidence level; e = margin of error (0.05); p = expected proportion (standard deviation) (0.5). From this calculation, the minimum sample determined was 375 first-to-fifth graders of 15,565 enrolled in elementary school, considering data from the 2021 School Census [35].

### 2.2. Acceptability Test

The resolution regulating the PNAE determines that schools conduct acceptability tests with students whenever a new menu is introduced, innovative changes to meal preparation are made, or evaluate the acceptance of frequently used menus [6]. This study defined acceptability as the acceptance index of the foods offered to schoolchildren, considering their sensory perception of characteristics such as flavor, texture, and appearance [31]. In this context, following the decision to remove chocolate milk—a commonly offered ultra-processed item—from school meals, milk with 100% cocoa and sugar was selected as the preparation for the acceptability test.

The milk with 100% cocoa and sugar was prepared by the lunch ladies so that the research did not alter the routine and standardization adopted in the schools. The time for the application of the acceptability test was defined with the school administration. In order to avoid doubts and errors during the application of the tests, 30 min before the application, visits were made to all selected classes to clarify the objective and procedures of the research, reinforcing and guaranteeing the participants of the freedom to refuse or withdraw their consent to participate in any phase of the research.

**Figure 1:**
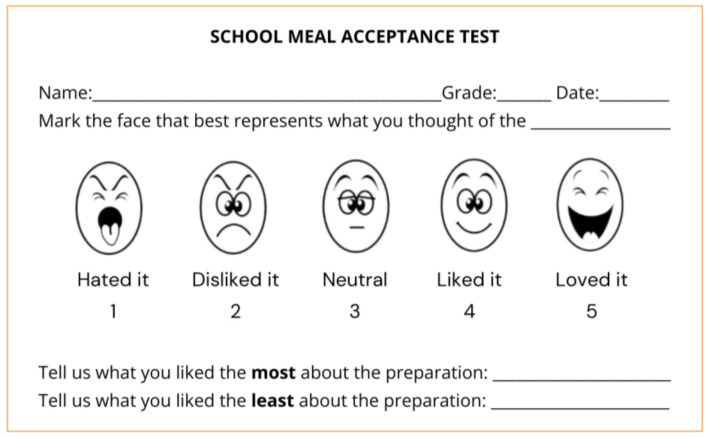
Five-point hedonic scale. Brazil. Ministério da Educação. Fundo Nacional do Desenvolvimento. Manual para aplicação dos testes de aceitabilidade no Programa Nacional de Alimentação Escolar. Brasília-DF, 2017 (public domain).

The affective-sensory acceptability test was conducted in the school lunchroom using a five-point hedonic scale (Figure 1) to assess students’ acceptance of a milk preparation containing 100% cocoa and sugar. Before starting the test, students were provided with a detailed explanation of the procedure, emphasizing its importance in assessing the acceptability of the preparation. Evaluation sheets were distributed, featuring a scale with five emotive facial expressions: “Loved it” (excellent), “Liked it” (good), “Neutral” (neither liked nor disliked), “Disliked it” (poor), and “Hated it” (very poor).

According to the Manual for Applying Acceptability Tests by the National Fund for the Development of Education (FNDE), when at least 85% of participants rate it positively, selecting the options “Liked it” or “Loved it” on the hedonic scale, the preparation is considered acceptable [31].

### 2.3. School Meals Consumption

A structured self-completion questionnaire was applied to evaluate the consumption of school meals in the classrooms of the selected classes, and it took an average of 15 min for students to complete. The questionnaire was based on the same manual [31] and divided into four blocks: (1) identification of consumption and school meals consumption frequency; (2) characteristics of the food served regarding the motivation for consuming school meals, amount, and temperature served; (3) food environmental conditions, which covers issues about the place available for meals, the time and utensils used; and (4) competitive foods. This last topic contained four questions: “Do you usually take food to eat at school?” When the answer was affirmative, there was space for the student to describe which food(s) they took. The foods described were tabulated and grouped into cookies, cake, sugary drinks (ready-to-drink juices and soft drinks in general) fruit, fruit juice, water, various sandwiches (burgers, bread with filling, and savory snacks), and treats (chocolates and sweets in general). The places where students usually buy these foods and the frequency they report when choosing them were also categorized. These questions allowed the study to target the accessibility and availability of competitive foods, providing insights into how these factors influence students’ food choices within the school environment.

School food consumption was established from the question, “Do you usually eat the food offered by the school?”. The answers were categorized as irregular consumption when the answer to the question: “How many days a week do you usually eat the food offered by the school?” was 1 to 3 days and regular consumption when it stated that it was consumed every 4 to 5 days, adapted from the model by Locatelli et al. [21].

### 2.4. Procedures and Data Analysis

A binomial logistic regression model was applied to assess the association between individual variables, school food supply conditions (exposure), and school food consumption (outcome). The significance level of 5% was used for associations. Analyses were performed using the Jamovi software, version 2.3 (2022). The analyses were conducted through the Jamovi 2022 software version 2.3.

### 2.5. Ethical Approval

All procedures involving human participants were approved by the Ethics Committee of Fluminense Federal University (registered under No. 98417718.5.0000.5243). All of the participants, as well as their parents or legal guardians, provided informed consent to participate in the study. They were included in the research through the Informed Consent and Informed Assent terms.

## 3. Results

A total of 1080 students participated in the study, of whom 51.6% (*n* = 558) were girls and 48.4% (*n* = 552) were boys. Of these, 630 students took the acceptability test, and 450 completed the questionnaire assessing school food consumption. Acceptance of the milk with 100% cocoa and sugar preparation, based on the frequency of votes on the 5-point hedonic scale, was 79.68%. Thus, this preparation was deemed not accepted (Table 1).

Regarding the consumption of school meals, most students (66.2%) reported consuming them, although irregular consumption (54.5%) was the most frequent pattern. Most of those who reported consuming (45.5%) consumed both meals offered (lunch and snack), and among those who consumed only one meal, the snack was referred to as the main one (39%). The main reason given for consuming school food was the pleasant taste (53.2%), and not liking the meal served (56.4%) on the day for not consuming it (Table 2).

Individual characteristics showed that not liking the lunch served at school increases the likelihood of not consuming school meals by 2.73% (*p* = 0.03). Bringing snacks from home showed no significant association with consumption (*p* = 0.677). The only characteristic of the school food supply that showed a statistically significant association with consumption was the cutlery used at meals. The inadequacy of this utensil increased the likelihood of not consuming school meals by 6.44% (*p* = 0.009) (Table 3).

In terms of the characteristics of the food served and its amount (*n* = 373), 79% (*n* = 296) stated that it was sufficient, while 17% (*n* = 63) stated that it was insufficient, and 4% (*n* = 14) said that it was too much. Repeating the meal (*n* = 386) was reported by 30% (*n* = 117) of the students. Concerning meal temperature (*n* = 371), the answers ranged from “sometimes it is good” (*n* = 186) to “it is always good” (*n* = 172) (50% and 46%, respectively), while the minority responded that “it is never good” (*n* = 13 and 4%).

Regarding environmental conditions (*n* = 263), 53.2% (*n* = 140) described the lunchroom as noisy, 22.8% (*n* = 60) stated that there was not enough space, 13.7% (*n* = 36) classified it as a dirty place, and 10.3% (*n* = 27) cited other reasons, such as a hot, cramped environment with uncomfortable chairs.

Concerning competitive foods, of the students who said they took food (from home or purchased around school) to eat at school (*n* = 350), 48% (*n* = 167) took cookies, 26% (*n* = 90) sugary drinks, 7% (*n* = 25) took fruits and sandwiches, 6% (*n* = 21) took sweets, 4% (*n* = 15) took cake and 1% (*n* = 4) took water and fruit juice.

As for the places where students purchased food, 22% (*n* = 85) reported purchasing it from the street market, 14% (*n* = 53) from bakeries, 51% (*n* = 199) from markets, and 13% (*n* = 50) from other locations. Regarding snack autonomy (*n* = 410), 46.8% (*n* = 192) reported going to commercial establishment with their guardians to choose snacks, while 35.6% (*n* = 146) stated they do not go, but have preferences. Only 17.6% (*n* = 72) responded that only those responsible chose the snacks.

## 4. Discussion

Replacing chocolate milk, commonly offered in school meals, with the preparation of milk with 100% cocoa and sugar resulted in an acceptance rate of 78.61%. However, it did not reach the minimum percentage of 85% required for the preparation to be considered acceptable. The acceptance below the minimum PNAE standards may be attributed to the fact that the replacement did not meet students’ flavor expectations, which likely influenced their negative perceptions. The preference for sweeter flavors, such as chocolate milk, which was common in school meals, may have contributed to this perception, suggesting that a less sweet and different flavor than usual may have generated student resistance, affecting their acceptance. The hyperpalatability of UPF has been linked to factors that promote and encourage their consumption [36].

A recent review analyzing the associations between the consumption of UPF and health outcomes in childhood and adolescence identified that the most frequent combinations of risk factors involved an unhealthy diet, with the regular consumption of UPF at the expense of a diet based on fresh or minimally processed foods, along with insufficient levels of physical activity. The authors concluded that these combined practices contribute to the increase in obesity prevalence and a sedentary lifestyle among children and adolescents [37].

Food provided under the PNAE offers multiple benefits for students, particularly through its positive association with the regular consumption of healthy foods [38]. In its most recent regulation enacted, Resolution N° 6/2020 [6] established limitations and restrictions on UPF to reduce the intake of these products in schools by providing food with better nutritional quality.

The results indicated that several factors influence the consumption of school meals, including individual preferences, food supply characteristics, conditions of the school environment, and the availability of competitive foods. These factors, along with others related to adherence to school meals, remain underexplored in the scientific literature, particularly in the context of national school food programs in Brazil. It is worth considering that other factors, such as the time at which meals are served and the size of the portions, although standardized to meet the nutritional needs of students, can influence the consumption of school meals.

While most students claim to consume school meals, irregular consumption remains prevalent, showing the need for interventions to increase regular consumption. It is essential to reach full participation to achieve PNAE’s objectives. A study conducted by Cesar et al. [19] also identified unsatisfactory consumption rates, with 66.2% of students in urban areas of southern Brazil consuming school meals, but 42.5% do so less than four days per week.

Among the factors associated with unsatisfactory consumption, individual food preferences, such as a dislike for certain meals, may be linked to unhealthy eating habits that are commonly related to the presence of unhealthy foods in the students’ eating context, including those associated with lower fruit consumption [39]. A national study with students identified a reported consumption of UPF one day before the survey by 97.3% of adolescents [40]. These foods are associated with adverse health outcomes such as obesity, metabolic syndrome, and dyslipidemia [41,42,43].

Although bringing snacks from home was not statistically associated with school meal consumption, a high percentage of students who brought snacks such as cookies and sugary drinks likely interfered with their willingness to consume school-provided meals. Andretta et al. [44] demonstrated that Brazilian students who buy or take snacks to school have a higher prevalence of UPF consumption. Similarly, Rossi et al. [28] found that more than half of the public school students in a Brazilian state reported consuming unhealthy snacks at school.

Various factors influence students’ food choices, shaped by their social and environmental contexts. These factors play a pivotal role in determining their dietary preferences and consumption patterns. Key determinants of food preferences and selectivity in children include biological aspects, eating behaviors, parental practices, and psychological, social, cultural, and economic factors, alongside the influence of social networks and marketing [3,45].

In this context, it is essential to address students’ vulnerability to marketing strategies for unhealthy foods, especially in commercial establishments and on widely accessed social media platforms [46]. Additionally, the food environment surrounding schools predominates the sale of UPF [23]. These factors can induce and facilitate the consumption and adoption of inappropriate eating practices that compete with the food offered at school.

Only the inadequacy of cutlery was associated with the outcome, although most classify them as adequate. This fact may be associated with students using spoons instead of forks to eat meals at school, which differs from how they eat at home, especially those in their final school years.

Many students raised issues of dissatisfaction with the meal offerings, describing the environment as dirty, hot, cramped, and noisy, with insufficient seats in the lunchroom and uncomfortable chairs. Considering that eating involves both taste and other senses, such as smell, touch, vision, and hearing, a pleasant environment can attract the individual, making eating a pleasurable and satisfactory moment [47]. Therefore, strategies to improve the ambiance of the school lunchroom can help make meals more attractive to students. In addition to the factors evaluated in this study, aspects such as school menu composition and acceptance of preparations offered can be included in future studies.

Carrying out the acceptability test with students is essential to guarantee a more attractive and nutritious school meal, which contributes to adopting healthy and sustainable habits among students and developing more effective strategies for promoting health and well-being in schools. Removing chocolate milk from school meals breaks with the tradition of its more frequent presence in the diet of students and professionals within the school environment. This change highlights the challenges associated with picky eating, which is characterized by an unwillingness to try new foods, as well as strong food preferences [48].

Eating habits acquired at home and enhanced by the COVID-19 pandemic, which affected access to adequate and healthy food and led to changes in eating patterns, can be one of the reasons that hindered the acceptability of milk with 100% cocoa preparation. During this period, there was an increase in the consumption of UPF [49], which generally have sweeter and more intense flavors, making it more difficult to adapt to more natural and bitter options [50]. Even when adding sugar to chocolate, ultra-processed cocoa drinks are still sweeter, which contributes to resistance to the more bitter taste of pure cocoa.

Permanent Food and Nutritional Education actions that involve the school community, including those responsible for students and municipal managers, can be strategic for improving the school food environment and increasing the acceptability of meals. These actions are essential for improving eating habits practiced at home, thereby promoting greater consumption of school meals. Engaging parents in this process is necessary, so that they also understand the importance of healthy eating since the students’ exposure to different eating routines prevents a proper understanding of what a healthy diet is. Furthermore, parents should monitor the food provided to their children and encourage them to eat various foods, serving as models to be followed, as there is a high probability that children will acquire healthy habits by mimicking parental behavior [51]. Families where parents know what a healthy diet is tend to have children who can make healthier choices. In contrast, children of busy parents who lack time for meals preparation more often have poor eating habits and tend to replicate their parents’ dietary mistakes [52], denoting the importance of the family environment in shaping the eating habits of the child and youth population.

Other means to improve acceptability and consumption can be planned by school food policy makers, such as strategies for menu innovations that include results from sensory testing with students.

This study highlights that merely adjusting PNAE legislation is insufficient; instead, strategies must be developed to improve menu acceptability and promote healthy eating within the school environment. Moreover, the identification of factors that can influence the consumption of school meals demonstrates characteristics that still need to be overcome by managers to increase students’ adherence to school meals, encouraging discussion to strengthen the PNAE. One limitation of this study is its focus on a single municipality, providing a localized perspective that may not fully represent other locations or contexts, thereby restricting the generalizability of the findings. Another limitation refers to the fact that an analysis based on the social situation, gender and age group of the students was not carried out.

## 5. Conclusions

The study identified acceptance below the minimum PNAE standards of milk with 100% cocoa and sugar, as well as the irregular consumption of school meals by students. These findings correspond to the proposed objective and indicate the need to seek more attractive alternatives to adapt students’ tastes to the demands of the proposed new menu, even if this involves an initially difficult acceptance of certain foods. However, it is important to note that the low acceptability may be influenced by the limited time this preparation has been offered. Students’ acceptance of this preparation might improve over time with more consistent and prolonged exposure.

Although the PNAE has updated its guidelines to ensure the supply of nutrient-rich foods and provide an adequate and healthy diet for students, school meals must also meet standards of flavor, variety, and temperature. Moreover, the school lunchroom and utensils must be adequate for the number of students to encourage greater consumption. The findings highlight the need for permanent food and nutritional education actions to emphasize the importance of school meal consumption for students’ health. Finally, stricter control over students’ snacks is essential to reduce the availability of competitive foods in these environments and promote greater adherence to school meals.

## Figures and Tables

**Table 1:** Frequency of total score on the hedonic scale of the acceptability test for the preparation of milk with 100% cocoa and sugar (*n* = 630). Brazil, 2023.

Response	*n*	%
(5) Loved it	406	64.44
(4) Liked it	96	15.24
(3) Neutral	54	8.57
(2) Disliked it	24	4.29
(1) Hated it	47	7.46
Total	630	100.00%
Acceptability		79.68%

**Table 2:** Characterization of school meal consumption reported by elementary school students (*n* = 450). Brazil, 2023.

Variables	*n*	%
School food consumption (*n* = 450)		
Yes	298	66.2
No	152	33.8
Meals consumed (*n* = 336)		
All (Snack and Lunch)	153	45.5
Lunch	52	15.5
Snack	131	39.0
Consumption classification (*n* = 355)		
Irregular consumption (1–3 days)	206	58.0
Regular consumption (4–5 days)	149	42.0
Reasons referred for consumption (*n* = 346)		
Pleasant Flavor	184	53.2
Healthy	97	28.0
Necessity/Hunger	65	18.8
Reasons referred for nonconsumption (*n* = 362)		
Not liking the food served on the day	204	56.4
Bringing snacks from home	158	43.6

**Table 3:** Association of individual conditions and school food supply with school food consumption reported by elementary school students. Brazil, 2023.

Individual Conditions and Meal Offer	School Food Consumption		OR	95% CI	*p*-Value *
	Yes *n* (%)	No *n* (%)			
Enjoy lunch (*n* = 346)	131 (37.9)	215 (62.1)			
Yes	104 (45.2)	126 (54.8)	Ref		
No	27 (23.3)	89 (76.7)	2.73	(1.41–5.28)	0.003
Dining hall condition (*n* = 416)	148 (35.6)	268 (64.4)			
Adequate	113 (38.6)	180 (61.4)	Ref		
Inadequate	35 (28.5)	88 (71.5)	1.103	(0.58–2.10)	0.766
Time to finish a meal (*n* = 415)	149 (35.9)	266 (64.1)			
Sufficient	80 (37.0)	136 (63.0)	Ref		
Short	69 (34.7)	130 (65.3)	0.88	(0.51–1.53)	0.649
Utensils used during meals (*n* = 345)	133 (38.6)	212 (61.4)			
Adequate	120 (40.3)	178 (59.7)	Ref		
Inadequate	13 (27.7)	34 (72.3)	0.719	(0.27–1.93)	0.514
Cutlery (*n* = 341)	131 (38.4)	210 (61.6)			
Adequate	126 (41.7)	176 (58.3)	Ref		
Inadequate	5 (12.8)	34 (87.2)	6.444	(1.60–25.95)	0.009
Queue (*n* = 381)	142 (37.3)	239 (62.7)			
Yes	94 (37.6)	156 (62.4)	0.893	(0.50–1.59)	0.702
No	48 (36.6)	83 (63.4)	Ref		
Brings snacks (*n* = 433)	145 (33.5)	288 (66.5)			
Yes	61 (28.1)	156 (71.9)	1.119	(0.66–1.90)	0.677
No	84 (38.9)	132 (61.1)	Ref		

* Statistical significance *p* ≤ 0.05; OR: odds ratio; CI: confidence interval.

## Data Availability

The original contributions presented in this study are included in the article. Further inquiries can be directed to the corresponding author.

## Abbreviations

PNAE	National School Meal Program
UPF	Ultra-processed foods
